# Italian Association of Clinical Endocrinologists (AME) and International Chapter of Clinical Endocrinology (ICCE). Position statement for clinical practice: prolactin-secreting tumors

**DOI:** 10.1530/EJE-21-0977

**Published:** 2022-01-06

**Authors:** Renato Cozzi, Maria Rosaria Ambrosio, Roberto Attanasio, Claudia Battista, Alessandro Bozzao, Marco Caputo, Enrica Ciccarelli, Laura De Marinis, Ernesto De Menis, Marco Faustini Fustini, Franco Grimaldi, Andrea Lania, Giovanni Lasio, Francesco Logoluso, Marco Losa, Pietro Maffei, Davide Milani, Maurizio Poggi, Michele Zini, Laurence Katznelson, Anton Luger, Catalina Poiana, AME

**Affiliations:** 1 Division of Endocrinology, Niguarda Hospital, Milan, Italy; 2 Section of Endocrinology and Internal Medicine, Department of Medical Sciences, University of Ferrara, Ferrara, Italy; 3 AME Scientific Committee, Milan, Italy; 4 Endocrinology Unit, IRCCS Casa Sollievo della Sofferenza Hospital, San Giovanni Rotondo (FG), Italy; 5 Neuroradiology, S. Andrea Hospital, NESMOS Department (Neuroscience, Mental Health, Sensorial Organs), Sapienza University of Rome, Rome, Italy; 6 Laboratorio Analisi Cliniche e Microbiologia, Synlab SRL, Calenzano, Florence, Italy; 7 Endocrinology, Ospedale Martini, ASL Città di Torino, Turin, Italy; 8 Pituitary Unit, Department of Endocrinology, Catholic University of the Sacred Heart, School of Medicine, Rome, Italy; 9 Internal Medicine 2, Treviso Hospital, Treviso, Italy; 10 IRCCS Istituto delle Scienze Neurologiche di Bologna, Bologna, Italy; 11 AME President, Endocrinology and Metabolism Unit, University Hospital S. Maria della Misericordia, Udine, Italy; 12 Department of Biomedical Sciences, Endocrinology Unit, Rozzano, Italy; 13 Department of Neurosurgery, Humanitas Clinical and Research Center IRCCS, Rozzano, Italy; 14 Endocrinology Unit, Bisceglie Hospital, ASLBT, Bisceglie, Italy; 15 Department of Neurosurgery and Gamma Knife Radiosurgery, IRCCS San Raffaele Scientific Institute, Vita-Salute University, Milan, Italy; 16 Department of Medicine (DIMED), 3rd Medical Clinic, Padua University Hospital, Padua, Italy; 17 Endocrinology, Department of Clinical and Molecular Medicine, S. Andrea Hospital, Sapienza University of Rome, Rome, Italy; 18 Endocrinology Unit, Azienda Ospedaliera S. Maria Nuova IRCCS, Reggio Emilia, Italy; 19 Department of Medicine, Stanford University Hospital, Stanford, CA, USA; 20 Division of Endocrinology and Metabolism, Medical University of Vienna, Vienna, Austria; 21 ‘Carol Davila’ University of Medicine and Pharmacy – Endocrinology, “C.I. Parhon” National Institute of Endocrinology – Pituitary and Neuroendocrine Disorders, Bucharest, Romania

## Abstract

Prolactinomas are the most frequent pituitary adenomas. Prolactinoma may occur in different clinical settings and always require an individually tailored approach. This is the reason why a panel of Italian neuroendocrine experts was charged with the task to provide indications for the diagnostic and therapeutic approaches that can be easily applied in different contexts. The document provides 15 recommendations for diagnosis and 54 recommendations for treatment, issued according to the GRADE system. The level of agreement among panel members was formally evaluated by RAND-UCLA methodology. In the last century, prolactinomas represented the paradigm of pituitary tumors for which the development of highly effective drugs obtained the best results, allowing to avoid neurosurgery in most cases. The impressive improvement of neurosurgical endoscopic techniques allows a far better definition of the tumoral tissue during surgery and the remission of endocrine symptoms in many patients with pituitary tumors. Consequently, this refinement of neurosurgery is changing the therapeutic strategy in prolactinomas, allowing the definitive cure of some patients with permanent discontinuation of medical therapy.

## 1. Introduction

### 1.a. Why this document

Prolactinomas are the most prevalent pituitary tumors. Medical therapy with dopamine agonists (DA) has been considered as the first-line treatment for relieving symptoms, normalizing hyperprolactinemia, and reducing tumor size in the majority of patients, whereas surgery as a second-line treatment indicated only for the minority of patients showing resistance or intolerance to DA, for patients with pituitary apoplexy, or based on patient’s preference (1). Technical advances have improved surgical outcome, obtaining total removal of the tumor, high rate of disease remission, and sparing normal pituitary function (2). Thus, the improvement of surgery could change in some prolactinomas the paradigm of DA chronic treatment as a first-line therapeutic strategy.

Prolactinoma is a clinical entity that includes different parameters: micro- and macroadenoma, males and females, patients with fertility consideration or postmenopausal, children or elderly patients, sensitivity or resistance to DA, presence of mass effect, and aggressive behavior (3). An individually tailored treatment is thus needed to manage a wide variety of settings, including the drawbacks of DA treatment in patients with psychiatric diseases.

To discuss all these aspects, a group of Italian experts has gathered together their clinical experience on the diagnostic and therapeutic challenges of prolactinoma patients, aiming to better define the most updated clinical approach and therapeutic options. Panelists were selected by the endorsing scientific societies (AME and ICCE) and included 15 endocrinologists with skill in the management of pituitary diseases (one skilled in scientific methodology), two neurosurgeons skillful in pituitary surgery and working in a high-volume pituitary center, one expert of laboratory medicine, and one neuroradiologist.

### 1.b. Methodology

Literature scanning has been performed in Pubmed via the MeSH terms coupled with free-text search. Bibliographic research has been completed using the similar articles function and following the authors of the most relevant publications. No filter has been selected for article type, publication date, language, or journal.

The grading of recommendations, assessment, development, and Eealuation (GRADE) system was adopted for the present position statement (4, 5, 6). In accordance with GRADE, evidence is categorized into four quality levels (high, moderate, low, or very low), while recommendations are classified as strong (‘recommendations’) or weak (‘suggestions’), on the basis of the quality of supporting evidence and level of agreement between the panel members (5). Whenever possible, the level of evidence (LoE) is reported in Table 1 using the following symbols: very low quality (⊗◯◯◯), low (⊗⊗◯◯), moderate (⊗⊗⊗◯), and high (⊗⊗⊗⊗). Briefly, ‘very low quality’ evidence is derived from unsystematic clinical observations (case reports, case series) or very indirect evidence (e.g. surrogate endpoints); ‘low quality’ evidence is from observational studies or randomized controlled trials (RCT) with major limitations; ‘moderate quality evidence’ derives from RCTs with significant limitations or from rigorous observational studies; ‘high quality evidence’ are well-performed RCTs and, in some exceptional cases, strong evidence from unbiased observational studies (5).

**Table 1:** Level of evidence (LOE) of the studies included for this statement.

Level of evidence	References
No LOE	1, 2, 3, 4, 5, 6, 7, 8, 9, 11, 12, 13, 14, 15, 19, 20, 22, 23, 25, 35, 36, 37, 38, 41, 51, 56, 60, 61, 64, 66, 67, 69, 71, 78, 79, 80, 82, 84, 85, 95, 97, 99, 100, 101, 102, 106, 107, 111, 113, 119, 128, 137, 149, 151, 152, 156, 158, 159, 162, 168, 176, 177, 179, 181, 187, 192, 197, 198, 200, 202, 205, 211, 213, 214, 215, 226, 227, 228, 229, 235, 236, 242, 243, 244
⊗◯◯◯	26, 31, 32, 33, 39, 40, 44, 45, 50, 52, 53, 54, 55, 62, 68, 70, 74, 75, 76, 77, 81, 83, 86, 87, 88, 89, 90, 91, 92, 110, 115, 116, 117, 127, 133, 150, 157, 174, 185, 189, 191, 193, 195, 196, 199, 204, 206, 220, 221, 232, 233, 234, 237, 239
⊗⊗◯◯	10, 16, 17, 18, 21, 24, 27, 28, 29, 30, 34, 42, 43, 46, 47, 48, 49, 57, 58, 59, 63, 65, 72, 73, 89, 93, 94, 96, 98, 103, 104, 112, 114, 118, 120, 121, 122, 123, 124, 126, 129, 130, 131, 132, 140, 141, 145, 146, 153, 154, 160, 166, 167, 169, 170, 171, 172, 175, 178, 182, 183, 184, 188, 190, 194, 201, 203, 207, 208, 209, 210, 212, 222, 223, 224, 230, 231, 238, 240
⊗⊗⊗◯	105, 109, 125, 135, 136, 138, 139, 142, 143, 144, 147, 148, 155, 163, 165, 173, 180, 216, 217, 218, 219, 225, 241
⊗⊗⊗⊗	108, 134, 161, 164, 186

In order to quantify the level of agreement among the panelists about the recommendations and suggestions, the RAND-UCLA appropriateness method has been applied (7). Briefly, for each recommendation or suggestion, the panelists have indicated a score ranging from 1 (maximum disagreement) to 9 (maximum agreement). Indications with median scores in the 1–3 range are classified as ‘inappropriate’, those in the 4–6 range as ‘uncertain’, and those in the 7–9 range as ‘appropriate’. Recommendations and suggestions rated as inappropriate have obviously not been released. Furthermore, some measures of the dispersion of panel ratings, which are taken as an indicator of the level of agreement/disagreement with which the ratings were made, have been calculated. Finally, for each recommendation/suggestion, the following additional information has been reported:

- Appropriate: panel median (range 7–9), without disagreement.
- Uncertain: panel median (range 4–6) OR any median with disagreement.

### 1.c. Epidemiology, morbidity, and mortality

Prolactinomas are the most common pituitary adenoma, accounting for approximately 50% of all pituitary adenomas, with a prevalence of ∼50 per 100 000 population and an incidence of 3–5 new cases/100 000/year (8, 9). In autoptic series, a high prevalence of undiagnosed pituitary adenomas (almost all tiny tumors) has been detected (10.4%) and prolactinomas represented 40% of them (10).

Based on tumor size, they are classified as microprolactinomas (microP, <10 mm diameter) or macroprolactinomas (MP, ≥10 mm diameter). MicroPs are mainly observed in premenopausal women, whereas MPs are more common in men aged more than 50 years. Giant tumors (>40 mm) are rare (1–5% of all prolactinomas). They are diagnosed mostly in men aged between 20 and 50 years (median 42) and occasionally in older men or postmenopausal women and even in the pediatric population, with a reported male-to-female ratio of ∼9:1 (11, 12).

In a few cases, other pituitary hormones are secreted in excess beyond prolactin (PRL) (mostly growth hormone (GH)).

PRL-producing pituitary carcinomas are rare and defined by the presence of cerebrospinal, meningeal, or systemic metastases (13).

In clinical series of patients aged more than 65 years, prolactinomas account for 4–8% of pituitary adenomas but the prevalence may be actually underestimated in elderly women and men due to reduced attention to symptoms of hypogonadism (9, 11).

Pituitary adenomas are rare in children and adolescents and account for 3–4% of all intracranial tumors in this age group (14). The mean age at diagnosis was 16.1 ± 2.5 years (range: 4.5–20 years). The incidence of prolactinomas in this age group is 0.1 per 1 000 000 population, and they are more frequently diagnosed in girls than in boys (up to 80% of cases) (15). MPs are more prevalent than microPs in pediatric populations (58% vs 42%) (16). Prolactinomas are larger and more frequently invasive in boys than in girls as in the adult counterpart (16).

Prolactinomas can also belong to genetic syndromes (see below at 4.d), with a reportedly higher aggressive behavior.

Prolactinomas are not associated with increased prevalence of diabetes, cardiovascular diseases, and cancer, as showed in a population-based cohort followed for 26 years in Tayside, Scotland (PROLEARS study) (17). Premature mortality was reported in patients bearing MP but not microP. The authors hypothesized that hypopituitarism-associated hormonal deficiencies or their overtreatment might contribute to adverse health outcomes.

## 2. Executive summary of recommendations (R)

All the R have been released with agreement, according to RAND-UCLA criteria (7).

### 2.a. Diagnosis (see sections 4.a for R 1–11, 4.b for R 2, 4.c for R 12–15, 4.d for R 16)

- **R 1.** We recommend measuring PRL levels in all patients with a clinical suspicion, namely oligo-amenorrhea in females, erectile dysfunction in males, galactorrhea or infertility in both sexes, and pathologic findings at MRI of the sellar region (level of agreement – LoA 9).
- **R 2.** We recommend a detailed medical and pharmacological history be taken. Physiological (pregnancy and breastfeeding), secondary, and iatrogenic causes of hyperprolactinemia should be ruled out (LoA 9).
- **R 3.** We recommend confirming the finding of randomly elevated PRL level with the insertion of an i.v. catheter and saline infusion for 15–20 min before blood sampling for PRL assay, unless PRL is clearly elevated (>80–100 ng/mL) (LoA 9).
- **R 4.** We suggest that any clinician should be acquainted about the employed PRL assays (unit of measure, etc) (LoA 8).
- **R 5.** We recommend screening for macroprolactin in (1) asymptomatic patients, (2) patients with atypical clinical picture, (3) patients with conflicting PRL results in distinct assays, and (4) patients with lack of decline of serum PRL levels with DA, and we suggest this screening in patients with macroadenoma and PRL levels in the so-called gray area (100–200 ng/mL) (LoA 9).
- **R 6.** We recommend that in cases of large pituitary adenomas (i.e. >3 cm) associated with normal or mildly elevated PRL levels, PRL levels should be measured after serial sample dilution to rule out hook effect (see text for details) (LoA 8).
- **R 7.** We suggest that the magnitude of PRL levels can be useful in determining the etiology of hyperprolactinemia in patients with pituitary tumors, namely that PRL levels >200–250 ng/mL are almost always due to a macroprolactinoma (LoA 8).
- **R 8.** We suggest defining a clear starting level of PRL (i.e. don’t settle for a vague indication of ‘higher than, for example, 1000 ng/mL’) in patients with MP to make easier the follow-up on pharmacologic treatment (LoA 8).
- **R 9.** We recommend screening of hypopituitarism at diagnosis (LoA 9):
  - In all patients with MP,
  - In microP only if there is a clinical suspicion.
- **R 10.** We suggest evaluating insulin-like growth factor I (IGF-I ) levels in all PRL-secreting tumors at diagnosis (LoA 7.5).
- **R 11.** We recommend that in cases of possible medication-induced hyperprolactinemia, high-field MRI with Gd should be performed only if there is persistence of hyperprolactinemia following withdrawal or replacement (whenever possible) of offending medication or if the patient is clinically unable to withdraw the drug suspected of causing the hyperprolactinemia (LoA 9).
- **R 12.** We suggest a careful neuroradiological evaluation guided by clinical context to avoid false positive diagnoses (LoA 9).
- **R 13.** We recommend MRI control within 3–6 months for MP and suggest it within 1 year for microP after DA initiation. Earlier follow-up should be considered in case of non-responders or new symptoms (LoA 9).
- **R 14.** We suggest limiting the use of Gd during follow-up (especially in MPs) (LoA 8).
- **R 15.** We recommend that a genetic basis for prolactinoma should be suspected based on family history, early onset of the adenoma (i.e. before 20 years) and aggressive behavior (i.e. uncontrolled tumor growth despite appropriate treatment), and concomitant other endocrine diseases (LoA 8.5).

### 2.b. Treatment

#### General aspects *(see sections 5.a, 5.b, 5.c, 5.f)*

- **R 16.** We suggest discussion of the therapeutic strategy in a multidisciplinary tumor board, mostly in MP (this should be a strong recommendation, but it was downgraded due to the limitations of real world) (LoA 8).
- **R 17.** We recommend referral to an expert pituitary surgeon (defined according to skill and caseload as detailed in the text) whenever neurosurgery is considered (LoA 9).
- **R 18.** We recommend that transsphenoidal resection of the adenoma be presented as a viable option to any patient with a radically resectable adenoma (microP or enclosed MP), ideally during a joint evaluation with the endocrinologist and the neurosurgeon (LoA 9).
- **R 19.** We suggest transsphenoidal resection of the adenoma whenever treatment is required and the patient is unwilling to take a chronic pharmacologic treatment (LoA 9).
- **R 20.** We recommend treatment with DA, namely cabergoline (Cab), at the lowest effective dose capable to control PRL hypersecretion and tumor volume (LoA 9).

- **R 21.** We suggest bromocriptine (Br) treatment in patients with intolerance to Cab who are not candidate for surgery, as well in the setting of Cab unavailability or low-resource countries (LoA 8).
- **R 22.** We recommend alerting patients who start Cab treatment and their caregivers about the possible development of impulse control disorders (ICD) and inquiring regularly for psychiatric symptoms during chronic treatment (LoA 8.5).
- **R 23.** We suggest periodic cardiac auscultation and ultrasonography in patients with a murmur or consuming more than 2 mg per week of Cab, without overlooking extra-endocrine causes of valvular involvement (LoA 7).

#### Microprolactinoma *(see sections 5.b, 5.c)*

- **R 24.** We recommend that the aim of treatment is the reversal of clinical picture, mainly hypogonadism (LoA 9).
- **R 25.** We suggest that simple clinical observation can be appropriate if hypogonadism or galactorrhea are not an issue, as well as oral estroprogestinic contraceptives in women not seeking pregnancy (LoA 8.5).
- **R 26.** We recommend surgery in patients resistant/intolerant to DA (LoA 9).
- **R 27.** We recommend DA withdrawal in females after the menopause (LoA 7.5).

#### Macroprolactinoma *(see sections 5.b, 5.c, 5.d, 5.f)*

- **R 28.** We recommend first-line Cab treatment, no matter how large the tumor size or severe the neurological and ophthalmologic damage, under tight clinical and lab control to quickly improve neuro-ophthalmologic symptoms (LoA 9).
- **R 29.** We recommend resection of the adenoma by an expert pituitary surgeon in patients without quick improvement of severe neuro-ophthalmologic damage on DA (within 2 weeks), or resistant/intolerant to DA, or with escape from DA effects (LoA 9).
- **R 30.** We recommend careful and tight follow-up of patients with partial response to DA and their quick referral to an expert neurosurgeon whenever the efficacy of DA treatment fades (LoA 9).
- **R 31.** We suggest that in good DA-responders (after PRL normalization and tumor shrinkage), follow-up can be safely performed in most cases only with PRL assessment at yearly intervals. MRI can be safely performed every other year or longer and even avoided if PRL levels remain normal (LoA 8).
- **R 32.** We recommend urgent evaluation by an ENT or a neurosurgeon in case of nasal cerebrospinal fluid (CSF) leakage (LoA 9).
- **R 33.** We suggest DA withdrawal in MP only in patients showing complete disappearance of tumor mass (or at least a 50% decrease in tumor size) and persistence of low-normal PRL levels after progressive downtitration of DA during chronic treatment, with a careful quarterly follow-up of PRL levels and gonadal status (LoA 7.5).
- **R 34.** We recommend against DA withdrawal in MP patients on chronic DA treatment with persisting tumoral tissue and pathologic PRL levels (LoA 9).
- **R 35.** We recommend irradiation in patients with MP that cannot be controlled by DA and surgery (LoA 9).
- **R 36.** We suggest that radiotherapy, regardless of the chosen technique, should be discussed within a multidisciplinary pituitary team (LoA 9).
- **R 37.** We suggest that, when available and technically feasible, radiosurgery should be used over fractionated radiotherapy, unless the tumor is too close to the optic pathways or tends to diffusely infiltrate the surrounding anatomical structures (LoA 9).
- **R 38.** We recommend prolonged yearly follow-up to evaluate the efficacy and safety of radiotherapy (LoA 9).

### 2.c. Special cases

#### Children *(see section 5.e.i)*

- **R 39.** We suggest DA treatment in children with prolactinoma, to preserve normal pituitary function, reserving neurosurgery for those resistant or intolerant to this treatment (LoA 9).

#### Gonadal replacement, contraception, fertility, pregnancy, and menopause *(see sections 5.b.iii, 5.e.ii, 5.e.iii, 5.e.iv)*

- **R 40.** We recommend early testosterone replacement treatment (TRT) in males with persistent hypogonadism, namely within 3–6 months after the start of DA, provided that PRL is progressively decreasing (LoA 9).
- **R 41.** We recommend a case-by-case evaluation of gonadal replacement therapy in hypogonadal women with MP (LoA 9).
- **R 42.** We suggest that hormone replacement treatment in hypogonadal female patients be continued at least until the age of physiologic menopause (LoA 9).
- **R 43.** We suggest estroprogestinic administration as a safe option in DA-responder women with microP or well-responsive MP requiring contraception (LoA 8.5).
- **R 44.** We suggest restoration of fertility with gonadotropins, whenever required (LoA 9).
- **R 45.** We recommend planning pregnancy (LoA 9).
- **R 46.** We recommend full information of the pregnant patient and easy access to an endocrinologic consultation (LoA 9).
- **R 47.** We recommend DA treatment (preferably Cab for better tolerability) while seeking pregnancy and its discontinuation at confirmation of pregnancy (LoA 9).
- **R 48.** We recommend against checking PRL levels during pregnancy (LoA 9).
- **R 49.** We recommend only clinical follow-up throughout gestation in patients with microP or a small intrasellar remnant of MP (LoA 9).
- **R 50.** We recommend clinical and pituitary function evaluation and neuro-ophthalmologic evaluation in patients with MP during pregnancy in each trimester (LoA 9).
- **R 51.** We recommend against pituitary MRI scan during uneventful pregnancy and in the early postpartum period (LoA 9).
- **R 52.** We recommend neuro-ophthalmologic evaluation, MRI without Gd, and pituitary function evaluation in patients who develop mass effect symptoms during pregnancy (LoA 9).
- **R 53.** We recommend reinstitution of DA during pregnancy (preferably Cab) in symptomatic patients with MP, to obtain a rapid remission of symptoms and to be maintained even after delivery (LoA 9).
- **R 54.** We suggest vaginal delivery in microP (unless otherwise indicated by the obstetrician) and a case-by-case evaluation in MP (LoA 9).
- **R 55.** We recommend that women with prolactinomas be allowed to breastfeed, provided that pregnancy was uneventful, postponing the possible restart of DA (LoA 9).
- **R 56.** We suggest adopting nonhormonal contraceptive measures after delivery or after the cessation of breastfeeding in order to evaluate PRL levels at 3–6 months in all women and subsequent MRI in MP (LoA 8).
- **R 57.** We recommend reinstitution of DA in women with relapsing symptomatic hyperprolactinemia after delivery (LoA 9).
  - **R 58.** We recommend DA discontinuation after menopause in microP, with yearly monitoring of PRL if elevated and MRI in case of progressive PRL increase (LoA 8).
- **R 59.** We recommend carrying on DA treatment in MP after menopause at the lowest dose capable to control tumor growth and follow-up according to clinical status (LoA 9).

#### Miscellany *(see sections 5.e.v, 5.e.vi, 5.e.vii)*

- **R 60.** We suggest that the treatment of prolactinoma does not change in the presence of breast cancer (LoA 9).
- **R 61.** We suggest evaluating PRL levels before starting treatment with neuroleptics (LoA 8).
- **R 62.** We recommend a tight collaboration between the endocrinologist and the psychiatrist for the management of patients with psychiatric disorders and hyperprolactinemia (LoA 9).
- **R 63.** We suggest that the patient with prolactinoma on antipsychotic treatment can be managed with DA with an individual evaluation of efficacy and safety (LoA 8).
- **R 64.** We suggest the evaluation of bone health to decide the additional start of bone-active treatment (LoA 9).

#### DA resistance and aggressive disease *(see sections 5.e.viii, 5.f)*

- **R 65.** We recommend tight follow-up in all patients with MP, mostly in males due to their elevated risk of unfavorable course (LoA 9).
- **R 66.** We recommend that an expert pituitary team adopt a quick multimodal approach (repeated surgery + radiotherapy + DA) in case of resistance or escape to DA or uncontrolled tumor growth (LoA 9).
- **R 67.** We recommend therapy with temozolomide in patients with MP resistant to DA who had unsuccessful surgical and/or radiation treatments or those with evidence of metastases (LoA 9).
- **R 68.** We suggest temozolomide withdrawal after the third cycle of treatment in patients with progression of disease (LoA 8.5).
- **R 69.** We suggest that patients with aggressive disease and no response to temozolomide can be treated with experimental approaches (LoA 8).

## 3. Clinical issues

### 3.a. Patients’ complaints that might lead to the evaluation for hyperprolactinemia

The patient may seek medical evaluation complaining of different troubles, according to gender and age.

Women in reproductive age complain of endocrine symptoms such as oligo-amenorrhea or short luteal phase (with more frequent cycles) (85–90%) (1, 8, 18), decreased libido (19), anovulatory infertility (20), and spontaneous or provoked galactorrhea. Galactorrhea occurs in nearly 90% of premenopausal women (21). Occasionally galactorrhea occurs in non-lactating women with regular menses and normal PRL values (‘nonpuerperal idiopathic galactorrhea’) (22).

Postmenopausal women usually present with symptoms due to mass effect related to a large tumor (see below), whereas galactorrhea is less frequent as the mammary glands are not primed with estrogen and progesterone anymore (21).

Approximately half of males typically present with symptoms caused by the tumor mass (see below) and the other half with symptoms of hypogonadism (8), most frequently loss of libido, erectile dysfunction, diminished ejaculate volume, infertility with impaired spermatogenesis, less frequently gynecomastia, and galactorrhea (23). All these effects are mostly caused through impairment of gonadotropin secretion and hypotestosteronemia (24). Due to hypogonadism, men may also complain of decreased energy and muscle mass, and anemia (11).

Clinical presentation varies by age and sex also in children and adolescents (16, 25). During the prepubertal period, headache, growth failure, and visual field defects are the most frequent signs, while during puberty, galactorrhea, hypogonadism, or pubertal arrest are additional characteristics.

Weight gain can be a presenting symptom for patients with newly diagnosed prolactinomas (26), even though its mechanism is still to be elucidated (27), and this should be a consideration in the investigative and treatment algorithm of prolactinomas. Overweight or obesity at diagnosis seems highly prevalent, both in pediatric (16) and adult age (28, 29). Weight loss is frequently recorded in prolactinoma male patients who normalized their PRL levels (29).

Patients at any age may seek medical attention due to mass effect symptoms occurring in about half of patients with MPs (8). The most frequent is visual impairment, due to upward adenoma extension/invasion with chiasmal compression, mainly expressing as field defects, such as unilateral or bilateral supero-temporal quadrantopia or temporal hemianopia. Headache is frequent in MP (but may occasionally occur even in microP) (30, 31, 32, 33). Hypopituitarism could be due to direct compression of the adenoma on the normal pituitary tissue or to stalk compression with hypothalamic disconnection, or even to apoplexy (8). In case of apoplexy, severe headache, visual impairment, and ophthalmoplegia may be the presenting symptoms.

Other symptoms depend on the site of adenoma extension/invasion. If it is upwards, it may occasionally cause hydrocephalus. In case of caudal extension, skull base structures destruction and occasional spontaneous CSF leakage may very rarely occur (34). Extension of the adenoma in other directions may cause other cranial nerve palsies (cranial nerves VI, IV, III), hearing impairments, hemiparesis, temporal epilepsy, or dementia due to frontal lobe extension (35).

### 3.b. Sequela of hyperprolactinemia

#### Osteoporosis

Hyperprolactinemia per se or through hypogonadism induces an increased bone turnover, with a predominance of bone resorption and, consequently, an increased occurrence of osteopenia and osteoporosis (11, 36, 37, 38). Suppressed levels of osteocalcin (OC), high collagen type I crosslinked N-telopeptide (NTX), and increased receptor activator of nuclear factor-kB ligand (RANKL)/osteoprotegerin ratio have been reported in patients with prolactinoma (39, 40). Both in females and in males, trabecular bone in the spine and in the hip is more affected than cortical bone in the distal radium; in fact, spinal bone mineral content is generally reduced from 20 to 30% and forearm bone mineral content from 2.5 to 10% (41). The difference in bone loss according to sites is likely due to a more rapid turnover of trabecular bone.

Tumoral hyperprolactinemia per se or through hypopituitarism may impair the attainment of peak bone mass in young patients (42) and bone mineral density (BMD) may be reduced in childhood-onset prolactinoma patients (43).

Hypogonadotropic hypogonadism is considered the main mechanism responsible for bone changes induced by hyperprolactinemia (44, 45, 46, 47). Some recent clinical studies have suggested that hyperprolactinemia can also act directly on bone. A higher prevalence of vertebral fractures has been shown in women and men with prolactinoma independently of gonadal status (48, 49). In a series of 78 women affected by PRL-secreting pituitary adenomas, the prevalence of vertebral fracture appeared to be related to the length and severity of hyperprolactinemia, to BMD T-score value, and to the treatment. Also, a 60% increase in the risk of clinical fractures in prolactinoma before the diagnosis of disease as compared to controls has been reported (50). On the other hand, the PROLEARS study showed an increased risk of fractures but only in the drug-induced hyperprolactinemia subgroup (17).

#### Quality of life (QoL)

Few data are available on QoL in patients affected by hyperprolactinemia and prolactinomas (51, 52, 53, 54, 55). Most of them are based on female population and on the SF36 questionnaire (a self-administered questionnaire that does not include sexuality and hypogonadism symptoms). Anxiety, depression, fatigue, and decreased well-being are the most frequently reported symptoms. Some studies have also reported sleeping difficulties. Despite usual QoL improvement after treatment, impairment of physical and emotional aspects as well as social isolation may persist. Data are discordant about the correlation between PRL levels and well-being awareness.

## 4. Diagnostic issues

The diagnosis of hyperprolactinemia is established by measuring basal PRL levels. Hyperprolactinemia is the hallmark not only of PRL-secreting tumors but also of non-secreting neoplasms of the hypothalamo-pituitary region and of a variety of disorders that must be excluded before prolactinoma is diagnosed.

The magnitude of PRL levels can be useful in determining the etiology of hyperprolactinemia. PRL levels generally correlate with prolactinoma size, namely up to 150 ng/mL in most patients with microP and higher than 200–250 ng/mL in patients with MP. An increase of PRL levels can be caused by non-secreting pituitary lesions (the so-called pseudoprolactinomas), such as non-secreting adenomas, as well as other tumoral, infectious, and inflammatory processes involving the hypothalamus, the perisellar region, the pituitary stalk, and the pituitary itself or a primary empty sella. This ‘stalk effect’ is due to the disconnection between the hypothalamus and the pituitary gland, with the consequent impairment of the inhibitory dopaminergic pathways and increase of PRL levels. In pituitary macroadenomas, PRL levels below 100 ng/mL can be attributed to this phenomenon, whereas PRL concentrations between 100 and 200 ng/mL represent a ‘gray zone’ that should prompt differential diagnosis between prolactinoma and pseudoprolactinoma (56, 57, 58, 59).

### 4.a. The conundrum of PRL assays and laboratory workup (R 1–10)

#### Pre-analytical requirements

PRL secretion is pulsatile, and levels are physiologically higher during sleep and in the early morning (60).

Emotional stress, venipuncture, exercise, walking, and a protein-rich diet all stimulate PRL secretion (61). Thus, specimens collected after an overnight fast, at least 2 h after awakening when the patient is resting, provide the most reliable PRL assessment (62). The insertion of an i.v. catheter 15–20 min before sampling for PRL assay in the diagnostic phase is a simple and practical tool in cases of mild hyperprolactinemia (63).

#### Analytical specifications

PRL assays typically involve noncompetitive, heterogeneous ‘sandwich’ techniques that use two antibodies that recognize different epitopes on the PRL polypeptide. PRL methods should be calibrated against reference materials with known international unit potency, such as the WHO first IRP 75/504, the second international standard (IS) 83/562, or the third IS 84/500.

Heterogeneity in the molecular size of PRL has been described with three major variants in the majority of sera from normal and hyperprolactinemic individuals (64). The monomeric or little PRL represents 80–95% of the total PRL in cases with normoprolactinemia and true hyperprolactinemia. The biological and immunological activity of PRL may be almost exclusively attributed to this form. Other forms of PRL with lower biological activity include the dimeric (big PRL) and the polymeric isoform or macroprolactin (big–big PRL), that account for less than 10 and 1%, respectively, of the total PRL levels in normal sera.

Macroprolactinemia represents a state of hyperprolactinemia characterized by the predominance of big–big PRL (at least 60% of circulating PRL), and it should be suspected in asymptomatic individuals or those without the typical hyperprolactinemia-related symptoms. Although the nature of macroprolactin is heterogenous, it is mainly recognized as an antigen–antibody complex, consisting primarily of monomeric PRL and immunoglobulin G isotype. The high molecular size of macroprolactin prevents its access to PRL receptors at the level of target organs, with the loss of hyperprolactinemia-related symptoms. The prevalence of macroprolactinemia ranges from 8 to 42% among hyperprolactinemic individuals (reviewed in (56)). Such patients should be reassured that neither pituitary imaging investigations nor DA treatments or follow-up are necessary.

Misdiagnosis and mismanagement were a frequent occurrence before the introduction of macroprolactin screening by the use of appropriate laboratory techniques (65), to determine the relative amounts of macroprolactin and monomeric PRL in blood samples of hyperprolactinemic patients, at least if clinical picture is not straightforward. The gold standard for the diagnosis of macroprolactinemia is gel filtration chromatography, but because this method is time-consuming and expensive, polyethylene glycol serum precipitation has been widely used as a screening method. A chemiluminescent immunoassay for monomeric PRL is now available, with lower interference with big–big PRL. Recoveries of PRL levels <40% are indicative of predominance of macroprolactin, whereas recoveries >60% point to the diagnosis of monomeric hyperprolactinemia (60). Anyway, the absolute value of monomeric PRL (either normal or elevated) should be considered for the diagnosis after removal of the polymeric forms (60). The assessment of macroprolactinemia has been recommended as financially justified since it reduces use of imaging and unnecessary and ineffective DA treatment in such patients (66). Macroprolactinemia associated with prolactinoma and typical hyperprolactinemic symptoms has been reported (67).

Artificially low PRL levels may result from the so-called hook effect. It is an assay artifact caused by an extremely high level of PRL, which saturates the detecting antibody used in the PRL sandwich assay, thus resulting in a falsely low reported value (60, 68). The hook effect may be unmasked by repeating PRL measurement after a serum sample dilution. This step will result in a dramatic rise in PRL levels if the patient has a MP, remaining unchanged in cases of non-functioning adenoma (60, 68). Hook effect is a very rare occurrence nowadays with modern assays and might be considered in all cases of large (≥3 cm) pituitary adenomas associated with normal or mildly elevated PRL levels (≤250 ng/mL).

Sample dilution should be considered when the laboratory report does not indicate a distinct PRL value, but only “level greater than, for example, 200 or 470 ng/mL (the levels corresponding to the upper value of the calibration curve for the most widely employed immunoassay platforms)", in order to set a clear starting level for follow-up on pharmacologic treatment.

Further assay interference should be considered and ruled out whenever there is discrepancy between measured PRL levels and clinical picture or MRI. Biotin is contained in many over-the-counter integrators and may cause interference in many assays that are based on biotinylated antibodies (69). PRL measurement should be repeated after the withdrawal of such interfering substances for a few days. Heterophil antibodies are another rare possible cause of misdiagnosis that could be overcome with appropriate treatment of the sample (70).

#### Endocrine workup in patients with prolactinoma

As in all pituitary macroadenomas, screening of hypopituitarism is warranted in MP. This should include in all patients the determination of morning serum cortisol and FT4, in order to start timely an appropriate replacement therapy (71). If not already available, testosterone levels in males and estradiol levels in females should be evaluated. The evaluation of gonadotropins can complete hormonal workup.

In case of microP, even though the occurrence of hypopituitarism was occasionally reported (72), an endocrine workup should be performed only if there is a clinical suspicion.

Regardless of tumor size, IGF-I evaluation should be performed at diagnosis in all patients with prolactinomas to rule out a mixed hypersecretion of GH and PRL (73). The search for other associated pituitary hormone hypersecretions should be driven by the clinical context.

### 4.b. The hunt for non-tumoral causes of hyperprolactinemia (R 2,11)

In many patients, increased serum PRL concentrations are due to physiological causes as pregnancy or stress, drug administration, or are secondary to diseases other than prolactinomas. PRL levels generally are only mildly elevated in these settings.

#### Secondary non-endocrine diseases

In liver cirrhosis, PRL is increased due to increased estrogen milieu and decrease of dopaminergic tone. Concentrations are generally lower than 100 ng/mL and are correlated to Child–Pugh score (74).

In chronic renal disease, decreased renal clearance and imbalance of neurotransmitters increase serum PRL in the range of 30–100 ng/mL (75). PRL levels can be even higher in the patient with chronic kidney disease consuming medications that interfere with dopaminergic inhibition of PRL. Only kidney transplantation restores normal PRL levels in patients with renal failure.

Increased concentrations of PRL have rarely been reported in the so-called ‘neurogenic’ hyperprolactinemias, such as breast manipulation (mammoplasty, piercing), chest wall disease (as Herpes Zoster), or neurinoma of intercostal nerves (11).

Another rare occurrence is severe PRL excess in association with internal carotid artery aneurism (76).

#### Endocrine diseases different from prolactinomas

The stalk effect due to the disconnection between the hypothalamus and the pituitary gland has been already described (see above).

The increase of PRL in primary hypothyroidism is well-known and correlates with the severity of hypothyroidism. Hyperplasia of thyrotroph and lactotroph cells due to TRH stimulation may induce a radiologically evident increase of pituitary size and a false diagnosis of pituitary adenoma in younger patients, in whom thyroxine replacement therapy normalizes pituitary size (77).

PRL levels are increased in 11–16% of patients affected by PCOS and 50–70% of these actually have a microP (8). The rise of PRL is generally mild but may contribute to hyperandrogenism (11).

#### Medication effects

Many drugs can increase serum PRL levels through different mechanisms. The most common and important action is through dopamine type 2 receptor (D2R) antagonism, others are inhibition of dopamine synthesis, dopamine depletion, and effects on serotonin metabolism or action (Table 2).

**Table 2:** Iatrogenic causes of hyperprolactinemia.

Antipsychotic drugs	First-generation or typical antipsychotics: phenothiazines, thioxanthenes, butyrophenones
	Second-generation atypical neuroleptic drugs: amisulpiride, risperidone
Antidepressant drugs	Tricyclic: imipramine, amitriptyline
	Selective serotonin reuptake inhibitors
Cardiovascular drugs	Reserpine, verapamil, α-methyl-DOPA
Gastrointestinal drugs	Metoclopramide, domperidone, l-sulpiride, cimetidine, ranitidine
Miscellaneous	Opioids, morphine, cocaine, marijuana
	Anesthetics
	Estrogens

Antipsychotic and antidepressant drugs are by far the most frequent (78). The occurrence and magnitude of PRL increase are dependent on the specific drug, but there is an individual susceptibility, due to age, sex, and D2R polymorphisms (79). The levels of PRL are generally lower than 100 ng/mL, but a few patients have higher values.

First-generation or typical antipsychotics (phenothiazines, thioxanthenes, butyrophenones) are characterized by a high potential to raise PRL levels. The second-generation or atypical neuroleptic drugs generally cause less frequent and marked increase of PRL, except for amisulpiride and risperidone. Furthermore, some second-generation antipsychotic drugs, such as aripiprazole, quetiapine, clozapine, ziprasidone, and olanzapine, have really low probability to increase PRL, and aripripazole can even lower PRL levels (80). With anti-depressant drugs the increase in PRL is generally mild, except for the selective serotonin reuptake inhibitors.

The antiemetics and prokinetics drugs domperidone, metoclopramide, and L-sulpiride determine a huge increase of PRL levels because of the antagonism at the D2R. This effect should not be overlooked as they are over-the-counter drugs.

Estrogens cause PRL increase (81), but this is less relevant with current dosages employed as contraceptives or replacement treatments. Most studies with estrogen replacement therapy (ERT) for menopause have shown either no effect or minimal effect on PRL levels with varying doses of estrogens up to 1.25 mg of conjugated estrogens or 50 µg of estradiol daily (60). About 20% of women taking combined oral contraceptives develop mild hyperprolactinemia (60), but it should be kept in mind the possibility of a concomitant organic cause for PRL elevation in cases of moderate-severe hyperprolactinemia (>50 ng/mL).

Whenever an iatrogenic cause of hyperprolactinemia is suspected, PRL measurement should be repeated after discontinuing the culprit medication for 3–4 days. If this is not feasible or safe (as in most patients on antipsychotic drug therapy), MRI should be performed to rule out a sellar mass (60).

#### Idiopathic hyperprolactinemia

In some patients with increased serum PRL levels, no specific cause is identified. Physiological conditions, endocrine and non-endocrine diseases, and drugs should be excluded. Macroprolactin should be excluded as well (see above at 4.a.) and pituitary MRI should be normal. The risk of subsequent development of a microP is very low (82).

Very few cases are familial and due to mutation of the PRL receptor (83).

### 4.c. Imaging of PRL-producing lesions when and how (R 11–14)

#### Technical specifications

MRI is the gold standard for the radiological diagnosis of prolactinoma. The basic evaluation of MRI comprises T1-weighted images (WI), T2-WI, and Gd contrast-enhanced T1-WI on coronal and sagittal planes. In some cases, when microP is not evident in pre-contrast imaging, dynamic T1 sequences with Gd are indicated (84). Although sensitivity for identifying microadenomas on 3-T MRI is reportedly better than on 1.5-T MRI in Cushing disease (85), no data support the same benefit in microPs. Thereby, there is no absolute indication to study patients with hyperprolactinemia with 3-T MRI. Additional sequences, such as susceptibility weighted images, particularly useful to assess blood and calcifications, also tagged as ‘T star (T*)’, may be useful in specific situations.

Only those patients with absolute contraindications to MRI will undergo CT scan. CT scan will demonstrate prolactinomas just like MRI but its accuracy in case of microadenomas is definitely lower. CT scan is usually performed, after MRI, before transsphenoidal pituitary surgery (TSS) to assess the anatomy of the sinuses.

#### Prolactinomas on MRI

Pituitary adenomas are usually mildly hypointense or isointense on T1-WI and have variable intensity on T2-WI (86). In one study, 80% of prolactinomas were reported to be hyperintense on T2-weighted MRI sequences when compared to the normal gray matter (87). Small cysts and hemorrhagic foci are common. Fluid–fluid levels can be present. T2 sequences can help recognize pituitary hemorrhage as well; this will usually appear hypointense in T2 both in acute and chronic stages, while cystic components will be hyperintense on T2 (88). Their response to therapy is similar to non-cystic ones (87, 89). Signal hyperintensity along the optic pathways on T2/FLAIR, due to edema, occurs in 15–20% of cases in which macroadenomas compress the optic chiasm. Most macroadenomas enhance strongly but heterogeneously on T1 post Gd. Subtle dural thickening (so-called dural ‘tail’), more typical for meningiomas, is present in 5–10% of cases. Many microadenomas appear slightly hypointense on T1 with Gd, others may enhance strongly and become isointense with the enhancing pituitary gland, being virtually invisible. Most microPs enhance more slowly than the normal pituitary gland. Between 10 and 30% of microPs are seen only on dynamic T1 post Gd imaging. If Gd is essential in the diagnosis of many microadenomas visible only after its injection, in macroadenomas it is able to better highlight features such as cavernous sinus invasion and position of residual normal pituitary gland.

One of the main problems when imaging a patient with suspect microP, especially with low hormone levels, is false-positive MRIs. False-positive MRIs can be related to the pathological interpretation of an anatomical variant or a technical artifact. These false positives are particularly dangerous, because they transform a normal subject into a patient who will undergo unnecessary follow-up and therapy. For this reason, it is essential that the MRI of the pituitary is interpreted by neuroradiologists, with an MRI of at least 1.5 T, and following a rigorous methodology that includes the previously defined technique.

In case of microadenomas, the main differential diagnoses are clinically non-functioning microadenoma, Rathke’s cleft cyst, or pars intermedia cyst. Main differential diagnoses of macroadenomas are pituitary hyperplasia, meningiomas, metastasis, craniopharyngioma, carcinoma, aneurysm, hypophysitis, and chordomas (90). Most of these pathologies can be easily differentiated from prolactinomas based on clinical symptoms, imaging features, and above all determination of PRL.

Pituitary adenoma invasion of local structures as assessed by MRI correlates with postoperative outcomes following surgical resection (91, 92, 93). The Knosp grade, used to evaluate adenomas extension into the cavernous sinus on preoperative MRI, has been shown to be predictive of intraoperative tumor invasion, total resection, and postoperative hormonal remission.

Pituitary hemorrhage is significantly higher in MP (20%) compared to microP (3%), regardless of DA therapy. It is most common in females. In most cases, it does not cause symptoms; its complete resolution takes several months (mean 26) (94). Subclinical hemorrhage is much more frequent than pituitary apoplexy. Pituitary apoplexy may be the first symptom of a previously undiagnosed pituitary adenoma and is often misdiagnosed until MRI is performed.

#### Follow-up imaging

There is no clear consensus on a precise timing of follow-up MRIs for prolactinoma patients. The Endocrine Society (ES) clinical practice guidelines suggest repeating MRI routinely within 1 year for microP and within 1–3 months for MP after DA initiation, according to clinical context (1). At variance, in MP in case of PRL levels rising despite treatment or new visual disturbances, headaches, or evidence of a new hormonal dysfunction, prompt follow-up is suggested (1).

Although there is a clear correlation between size of adenoma and PRL levels (57, 58, 59) before and after treatment, there are cases of discordance between tumor changes and PRL levels during therapy (95). In most of these rare cases, enlargement of MP during DA therapy with stable/reduced PRL levels is mostly related to pituitary hemorrhage (96). The timing of further MRI follow-up after the first performed at 1–3 months should be based on the individual clinical context, including the changes of neuro-ophthalmologic and endocrinological picture, pre-treatment adenoma size, signs of invasiveness, prior surgery, rate of PRL decline and tumor shrinkage on DA treatment, sex, estrogen state, as well as adherence to the medication (97). During chronic DA treatment, it is extremely important to review the entire series of available MRIs because subtle changes may not be observed when a comparison is limited to just two consecutive studies.

Enlargement of microP while on DA therapy was reported to be extremely rare (96). This observation has led some experts to recommend not performing follow-up MRI at least for microPs (98) unless PRL rises significantly (e.g. >250 ng/mL) or if severe headache, impairment of visual fields or visual acuity, or cranial nerve palsies develop (99, 100).

Because of the well-known deposition of most Gd chelates in many tissues (101) and the debate regarding the long-term effects of Gd exposition, it is suggested avoiding Gd-enhanced MRIs in the prolonged follow-up of MP.

### 4.d. When to suspect and how to screen for genetic diseases (R 15)

Most prolactinomas are sporadic as the other types of pituitary adenomas. Nevertheless, 1.5–3% of cases have a familial basis.

All patients with prolactinoma need a careful family and personal medical history considering the suspect of a hereditary form. Early onset of the adenoma and aggressive behavior are additional elements to suspect a genetic form.

Multiple endocrine neoplasia (MEN) type 1 (MEN-1) is an autosomal dominant syndrome with high penetrance in which prolactinoma is the most frequent pituitary adenoma, present in 40% of patients (though pituitary adenomas in MEN-1 represent less than 3% of all pituitary adenomas). Serum calcium should be measured to rule out primary hyperparathyroidism, the most common manifestation of MEN-1. Screening for pituitary tumors should be started at 9 years of age in carriers of the menin mutation (102). Prolactinomas in MEN-1 were traditionally considered more aggressive than sporadic ones, but recent studies based on early screening did not confirm such difference (103).

Familial isolated pituitary adenoma is characterized by the presence of only pituitary adenomas in affected members. Aryl hydrocarbon receptor interacting protein(AIP) is the most frequently involved gene. Transmission is autosomal dominant with relatively low penetrance. Prolactinomas along with somatotropinomas are the most frequent type of adenomas and show an aggressive behavior (104).

In Carney complex and in X-linked acrogigantism (X-LAG) syndrome, PRL is frequently increased but the clinical characteristics are those of somatotropinomas due to GH excess.

No prolactinoma has been reported in MEN-4.

## 5. Therapeutic issues

### 5.a. Aims of treatment

The ideal treatment for prolactinomas depends on its etiology:

- In microP, it aims for restoration of eugonadism and fertility and the resolution of galactorrhea.
- In MP, the aims are tumor shrinkage with disappearance of neurological and ophthalmologic symptoms, normalization of hyperprolactinemia with the consequent restoration of eugonadism and fertility, and the resolution of galactorrhea.

### 5.b. Pharmacologic treatments

#### 5.b.i. Dopaminergic drugs (R 20–23, 27–31, 33–34)

Medical therapy with DA represents the first-line treatment in the management of almost all patients with prolactinomas, including microadenomas, macroadenomas, and giant adenomas (1).

DA normalizes serum PRL levels in almost 90% of patients with idiopathic hyperprolactinemia or microP and in 75–80% of patients with macroadenomas (105). Tumor shrinkage has been observed in more than 90% of treatment naïve MP patients (57).

Both hormonal and tumoral effects are mediated by the binding of DA to D2R on the membrane of adenoma cells, leading to reduction in synthesis and secretion of PRL and shrinkage of adenoma cells up to apoptosis (106).

Available DA are Cab, Br, quinagolide, pergolide, and metergoline. Pergolide and quinagolide are not available in Italy, metergoline is used only rarely.

Cab is the first choice of DA due to its efficacy, long-acting effect (half-life ranging 63–109 h), and relatively uncommon adverse events (107). Cab was shown to be superior to the first used DA Br, due to its greater efficacy in decreasing and normalizing PRL levels, reducing tumor size, and its better tolerability (108). The bioavailability of Cab is not influenced by food intake (107). In general, Cab is started at 0.25–0.5 mg weekly and given once or twice a week after dinner or at bedtime. According to clinical picture, the dose is uptitrated if needed at 1–3-month intervals in microP, at weekly intervals in MP with visual impairment (109).

Some patients are still treated with Br in specific situations, such as patients well controlled on Br for many years, or those that do not tolerate Cab, or anecdotal cases of resistance to Cab (110). Br is conventionally given in two or three daily doses; however, a single evening dose has been shown to be equally effective. To prevent adverse effects, it is advisable to start treatment with a low dose during the evening meal or at bedtime (1.25 mg) and gradually increase the dose by 1.25 mg every 2–7 days (111); doses greater than 30 mg rarely have been used.

##### Microadenomas

The aim of the treatment is to revert the effects of hyperprolactinemia, that is, spontaneous galactorrhea, and to restore gonadal function, that is, ovulatory menses in females, normal testosterone levels and sexual potency in males, and libido in both sexes. Treatment should be offered also to regularly cycling females with pathological hyperprolactinemia and anovulatory menses desiring pregnancy, and males with pathological hyperprolactinemia and normal testosterone levels complaining erectile dysfunction. Tumor shrinkage is not an issue in this setting because significant or persistent growth is uncommon according to studies of natural history of untreated microPs (112), even in the presence of local tumor invasion. Amenorrheic premenopausal women not desiring pregnancy may be treated only with oral contraceptives, without DA, provided that patients do not complain of symptoms suggestive of tumor size increase and/or galactorrhea, PRL levels do not substantially increase, and evidence of tumor enlargement is not observed while on this treatment (1).

In females, Cab should be administered at the lowest dose capable of restoring regular menses and suppressing galactorrhea. The clinical response occurs within 12 weeks in about 80–90% of patients. In the steady state, this target is usually maintained with 0.25 mg twice a week or 0.5 mg once a week, according to tolerability or the patient’s preference. Female patients should be informed that treatment can early restore ovulation. Contraception may be thus required if needed. In a review of 14 prospective studies in patients with hyperprolactinemic disorders, Cab was successful in normalizing PRL levels in 73–96% (113). In addition, tumor shrinkage was reportedly observed in 50–100% of Cab-treated microP (113).

A wide variability exists in clinical response. Some females require full PRL normalization to resume ovulatory menses, whereas in others, clinical response may be observed despite still pathological PRL values. At variance, in males usually testosterone levels are normalized and sexual activity is fully regained only after the normalization of PRL levels that may require several weeks. Accordingly, Cab dose should be individually tailored, with uptitration to 1.0–2.0 mg/week (or even more) or downtitration to the lowest effective dose.

DA should be discontinued in pregnancy (see below at 5.e.iii) and might be withheld in some particular cases, such as perimenopausal women, asymptomatic postmenopausal women, or asymptomatic men without hypogonadism in whom simple observation with periodic monitoring of PRL could be considered (114, 115, 116, 117).

##### Macroprolactinomas

The aims of the treatment in these patients are the quick relief of neuro-ophthalmologic symptoms when present, the normalization of PRL levels, and tumor shrinkage. The first is a compulsory aim in all patients, whereas the second and third ones should be pursued but are not always obtained.

Cab is always the first-line option in MP, even in patients bearing huge adenomas and/or severe neurologic symptoms or visual defects. In these patients, the thorough uptitration of Cab can quickly improve neurological and visual symptoms and lower abruptly PRL levels.

Cab is effective at doses ranging from 0.5 to 2 mg weekly in most MP (118). A marked decline in PRL levels and tumor shrinkage may be obtained with very low Cab dose (0.5 mg/week) even in patients bearing very large adenomas and very high PRL levels.

Responsiveness to DA is very common but cannot be predicted with certainty (119). The attempt to create retrospectively a predictive score combining demographic, biochemical, and tumoral parameters was unsuccessful (120). Accordingly, it is worthwhile to begin treatment and monitor PRL values and tumor size. It has been reported that the first PRL values obtained during treatment (either evaluated as absolute value or as percent decrease from baseline or as lowering below a predefined threshold, according to different series) as well as early tumor shrinkage (with different cut-offs in different series) are good predictors of long-term DA efficacy (120), either evaluated at 6 months (121) or even earlier (122, 123, 124). On the contrary, it was recently reported but not yet confirmed that heterogeneity of prolactinoma T2 signal at diagnosis could be used as a negative predictor factor of hormonal response to DA (87).

In responsive patients, Cab achieves progressive PRL decrease and tumor shrinkage for years, reaching hormone normalization and tumor shrinkage up to its disappearance or empty sella in most. In these highly responsive patients, Cab dose may be progressively lowered (to 0.25–0.5 mg/week or even administered at longer intervals). On the other hand, a stepwise dose uptitration may be necessary in some resistant patients in whom the average Cab dose does not achieve hormonal and tumoral targets (125). An extreme uptitration (up to 7–12 mg/week) is anecdotic. It is reasonable to continue with further dose increase whenever this is followed by a substantial PRL decrease, whereas it is recommended to go back to the lowest dose that caused the lowest PRL levels without increase in tumor size, in order to avoid side effects.

Some patients experience an extremely rapid decrease in tumor size with a significant improvement in visual fields within 24–72 h. Improvements in visual fields generally parallel the changes observed at imaging, whereas reductions in PRL levels usually forerun any tumor shrinkage.

In MP patients, there is usually a positive correlation between PRL levels and adenoma size. In most cases, PRL reduction and tumor shrinkage follow a parallel course during Cab treatment, although there are exceptions: in some patients PRL decrease is faster than tumor size reduction, in others PRL decrease is accompanied by an improvement of visual fields without a significant change of tumor volume. In very rare cases, PRL levels decrease in spite of tumor size increase (95).

Patients with persisting neuro-ophthalmologic symptoms, or tumor size increase/reincrease during treatment, or PRL reincrease despite carrying on treatment (‘escape phenomenon’) should be referred to neurosurgery (see below at 5.c).

**Follow-up**. In most severe cases, controls should be performed at short intervals:

- Neuro-ophthalmologic examination and clinical evaluation should be tightly evaluated within the first month in order to guide timing of neurosurgical interventions when needed.
- PRL levels should be evaluated weekly or monthly for the first 3 months and then at longer intervals if treatment is effective.
- MRI control should be performed according to ophthalmologic and PRL changes on treatment.

In less severe cases without visual impairment, biochemical, ophthalmologic and neuroradiological follow-up should be less aggressive, with the first evaluation after 3–6 months and the following according to clinical course: every 6–12 months in responsive patients and more tightly in partially responsive patients (3–6 months).

In patients whose PRL levels and tumor size are progressively reduced on DA treatment, the efficacy of treatment can be maintained even when DA dose is tapered.

##### Side effects, intolerance to treatment, and precautions to be taken (R 21–23)

Most adverse events of DA are mild or moderate in severity.

The most typical side effects of DA are nausea, vomiting, postural hypotension, drowsiness, somnolence, nasal stuffiness, headache, Raynaud’s phenomenon, and constipation. Nausea and postural hypotension occur frequently with Br, even at the lowest doses at the beginning of treatment, and require shifting to Cab.

Cab is generally better tolerated than Br, any side effect usually subsides over time but in a few cases (3–4%) they may persist, requiring Cab downtitration or withdrawal and referral to neurosurgery.

Care should be exercised when administering DA concomitantly with other medications known to lower blood pressure. DA should be used with caution in patients with a history of neuropsychiatric disease.

DA-induced neuropsychiatric symptoms may be much more worrisome, including psychosis (or exacerbation of pre-existing psychosis) and ICD, such as compulsive gambling, shopping or eating, and hypersexuality, which resolved after either DA reduction or cessation (126, 127, 128, 129, 130). These features can have devastating effects on the patient and his/her social environment. The exact incidence of DA-induced psychosis occurrence or exacerbation is not known but likely to be less than 1% (56), whereas the true prevalence of ICD remains unclear. In an uncontrolled study of 308 patients with prolactinoma treated with Cab for at least 3 months, 17% developed an ICD (131), a figure higher than in patients with non-secreting pituitary adenoma and no history of DA therapy and far lower than in patients with Parkinson’s disease who usually are treated with higher doses of Cab. No association with DA type, dose, or duration of treatment was noted. A recent Australian paper evaluated specific neuropsychological questionnaires in 113 patients with hyperprolactinemia (prolactinoma in 95%) and 99 normal controls and reported a 50% increase of relative risk in patients (up to 60%) compared to a surprisingly high prevalence of ICD in the control group (40%) (132). It has been suggested that patients (and family members/caregivers) should be forewarned of the possible development of ICD while on DA therapy.

In patients bearing large invasive MP extending through the sellar diaphragm and eroding the sellar floor, CSF nasal leakage has been rarely reported during DA treatment, either within the first few weeks or later after several months (34). This extremely severe complication is due to rapid tumor shrinkage that allows the leakage of CSF through an emerging tumor-induced gap in the skull base. The patient should be alerted to seek medical advice in case of watery nasal discharge. Analysis of fluid for beta-2 transferrin or beta trace protein is a well-known, specific method for detecting the presence of CSF (133) but unfortunately it is not largely available. CSF leakage should prompt urgent evaluation by an ENT or a neurosurgeon (134).

Cab and pergolide (but not Br due to its lower affinity to serotonin 2B receptor) have been associated with valvular heart disease in patients with Parkinson’s disease (135, 136, 137). A recent meta-analysis observed a statistically significant increased risk of tricuspid valvular dysfunction with the use of Cab but in no patient tricuspid valve dysfunction was diagnosed as a result of clinical symptoms. In addition, there was no significant increase in any other valvulopathy (138). Neither treatment duration nor cumulative dosage was associated with an increased risk of tricuspid valve lesions of any severity and the clinical significance of these findings is therefore questionable. The use of the lowest effective dose of Cab and serial cardiac ultrasonographic examinations are suggested in patients taking larger doses of Cab. Current data do not support major concerns about the risk of valvulopathy in hyperprolactinemic patients who are chronically treated with DA at standard doses (≤2 mg/week) (139). Subclinical valvular abnormalities detected by ultrasonography are not an indication for discontinuation of DA treatment. If valve lesions are detected during follow-up, further evaluation is indicated to distinguish Cab-induced etiology from other causes of valvulopathy.

#### 5.b.ii. How long to treat (R 33, 34, 58, 59)?

A major drawback of DA therapy is the potential need to keep the medication indefinitely in many patients. The optimal treatment strategy and duration of treatment is still not evident. It has been reported that starting treatment with high Cab doses to obtain a faster PRL normalization and tumor shrinkage is the best way to obtain disease remission (140), but data are still scanty on this topic.

In 2003, a landmark study (141) demonstrated that Cab could be withdrawn in a considerable proportion of selected patients. Recurrence of hyperprolactinemia and increase in tumor size were reportedly variable in several following studies after DA discontinuation. In a metanalysis of 19 studies with a total of 743 patients, the overall remission rate after DA withdrawal (defined as persistent normoprolactinemia after a 7–57-month follow-up) was only 21 and 16% for microP and MP, respectively (142). Another more recent metanalysis (143) showed that long-term disease remission rate in microP premenopausal women after the withdrawal of any DA was 36% (95% CI, 21–52%) and after the discontinuation of Cab was 32% (95% CI, 18–48). The remission rate was even lower in MP (28%, 95% CI 8–51%).

Different studies reported that different factors are able to affect positively the success rate of DA withdrawal, such as the rate of PRL decrease after the start of DA treatment (120), tapering Cab dose before withdrawal (144), low-dose (0.5 mg/week) maintenance therapy for at least 1 year, restoration of a normal serum PRL level, and a significant reduction in tumor size or its disappearance (142, 143, 144). On the contrary, Cab treatment duration longer than 2 years was not a positive predictor (144) and parasellar invasion was a negative predictor (120).

In patients with MP guidelines from ES suggested that Cab could be withdrawn if normal PRL levels are maintained for at least 2 years despite progressive lowering of Cab dose and no visible tumor remnant on MRI (1). PRL levels should thereafter be monitored every 6–12 months to detect any later recurrence.

Some papers previously reported about the attempt of DA withdrawal after a first unsuccessful trial, with a success rate ranging 18–30% (145, 146).

From a practical point of view, Cab treatment should not be withdrawn if PRL levels reincrease after lowering Cab dose, and the management should be different according to sex and tumor size.

- In microP, treatment should be lifelong in male patients to maintain normal sex hormones, whereas in females, it can be safely stopped after the menopause, when estrogen fall leads to the spontaneous normalization of PRL, allowing to stop prolonged follow-up in patients with a long history of PRL normalization.
- In MP, treatment should be lifelong and the attempt of withdrawal is only occasionally successful but requires anyway follow-up. The surgical option can be considered to obtain remission and allow the patient to get rid of any treatment.

After an attempt of withdrawal, PRL should be measured at 3 months and thereafter according to this first result. After withdrawal of DA therapy, the decision to repeat MRI may rely mostly on the degree of PRL elevation, arbitrarily set after a cut-off of 100 ng/mL.

The delicate balance between the cost-effectiveness of a simple yearly monitoring of PRL levels on a minimal DA dose on the one hand and a more intensive biochemical and neuroradiological monitoring in the attempt to withdraw the treatment in patients with a MP remnant on the other hand should be individualized, taking into account also the impact of both strategies on QoL.

#### 5.b.iii. Restoration of gonadal function and fertility when and how (R 40–42, 44)

Hypogonadism is often present at diagnosis of PRL-secreting adenoma as PRL regulates gonadal steroid secretion. Chronically elevated PRL levels and hypogonadism are associated with reduced BMD and osteoporosis (45, 46). Moreover, untreated hypogonadism increases mortality in both sexes, due to cardiovascular diseases, while gonadal replacement therapy restores standard mortality rate (147, 148).

Even though DA very often obtain reversal of hypogonadism, especially in microP and MP with normal TSH-thyroid and ACTH-adrenal function (149), gonadal replacement therapy may be necessary in patients with persistent hypogonadism despite lowering/normalization of PRL levels or being resistant to DA.

##### Premenopausal women

In spite of estrogen sensitivity of lactotrophs, in microP patients using oral contraceptives or ERT, tumor size rarely increases even without DA treatment (149, 150, 151). Women with microP who do not require fertility restoration may be treated only with estroprogestinic preparations (or estrogen alone in hysterectomized women) rather than DA.

Tumor growth is more concerning in patients with MP. Therefore, in cases with persistent amenorrhea a thorough case-by-case evaluation is required, with very close monitoring of tumor size and PRL levels if estrogen therapy is prescribed (151), due to the potential estrogen-induced decrease of DA efficacy. ERT should be continued at least until the age of physiologic menopause or longer evaluating the individual risk profile, taking into account the risk of osteoporosis.

DA treatment restores fertility in most women, even before menses occur (152). When normal menses are not restored, recombinant gonadotropins may be used for ovulation induction (149).

##### Males

In males, impaired BMD and anemia are the main consequences of long-term PRL-induced hypogonadism. Commonly, normalization of PRL and testosterone levels improves anemia as well as other manifestations of hypogonadism and is associated with bone health status improvement (153).

There are not enough data regarding predictors of long-lasting/persisting hypogonadism. In a recent retrospective study with a follow-up of only 2 years, hypogonadism persisted in 74% of patients after PRL normalization with Cab; the higher PRL levels and tumor size, the lower the chance of normalizing testosterone levels (154). In another study, long-term treatment (median: 3 years) was necessary to obtain normalization of hyperprolactinemia and reversal of hypogonadism (155).

Even though there is a potential risk that testosterone aromatization into estrogen could stimulate proliferation and hyperplasia of prolactinoma cells, thus inducing DA resistance (156), this occurrence is very rare (157), and the usefulness in this setting of aromatase inhibitors as add-on treatment was reported (158). TRT is thus indicated in case of hypogonadism persistence (159). Reports on the adequate timing for starting TRT are missing. Clinical experience suggests that in patients with pituitary failure, age-adjusted TRT should be quickly added to adrenal and thyroid replacement therapies, in order to improve clinical conditions and restore normal libido. As for isolated hypogonadism, an individual evaluation should guide the start of TRT. Given all these data, an early TRT may be suggested (within 3–6 months after the start of DA), provided that PRL levels are progressively decreasing and tumor size is shrinking.

TRT should be started soon also in patients partially sensitive to DA. In these cases, a tighter control of PRL levels (or MRI if needed) is suggested.

In male patients, treatment with clomiphene citrate or gonadotropins may be considered to restore or induce fertility, respectively. Clomiphene citrate increases serum testosterone levels and improves sperm motility, even in the absence of normal PRL levels (160).

### 5.c. Neurosurgery feasibility and appropriateness (R 17–19, 26, 29, 30, 32)

With the discovery of DA efficacy and the commercialization of Br, medical therapy became the treatment of first choice for prolactinomas. In the ’90s, medical therapy improved with Cab introduction (161).

Surgery was traditionally indicated as a second-line treatment in 14–38% of prolactinoma patients (162) fitting certain conditions such as:

- resistance or escape to DA,
- intolerance to DA,
- in some psychiatric disorders worsened by DA treatment,
- spontaneous or DA-induced CSF leakage,
- patient’s preference.

In the last two decades, a significant refinement in imaging and surgical techniques, particularly in TSS, has been attained in high-volume centers. Both microscopic and endoscopic pituitary surgery are minimally invasive techniques available for surgical treatment of pituitary adenomas (163). They have equally low complication rate and high cure rate. A recently published meta-analysis (164) shows that long-term disease remission is obtained in 74% of patients after surgery alone vs 37% after medication withdrawal. In microP, patients long-term remission was 36% after medication withdrawal and 83% after surgery. In MP, 60% of patients experienced long-term remission after surgery, compared to 28% after medical therapy withdrawal. Surgical results were not influenced by the surgical technique. These data mean that almost 60–70% of patients under DA treatment cannot discontinue their medical therapy.

Literature published in the past 15 years shows favorable rates of postoperative normoprolactinemia in 71–100% of enclosed adenomas, particularly in microP (2). Remission rates ranges from 71 to 93% for microscopic series and from 82 to 100% for endoscopic series (2). Regarding MPs, the results are particularly good in enclosed adenomas, with early remission in 95% and long-term remission in 89% of the cases (165, 166).

The cure rate dramatically drops with the invasive character of prolactinomas (167). Endocrinological remission is inversely related to adenoma size, and it is 10% for giant or invasive tumors (168). Invasion of the cavernous sinuses and the highest level of preoperative hyperprolactinemia are the two best factors to predict a poor endocrinological outcome after surgery.

Considering both microP and MP, mortality in experienced hands in high-load centers is nearly 0% and major complications occur between 1 and 4% of patients (169) (Table 3).

**Table 3:** Complications after neurosurgery for prolactinomas.

Reference	Patients, *n*		Acute complications									^†^Hypopituitarism
	Total	microP	Death	Visual worsening	Transient DI	DI	SIADH	CSF leak	Meningitis	Other (microP)	^*^Total (microP)	
(170)	120	59	0	1.7%	-	-	-	-	-	3.3% (0.6%)	5% (0.6%)	3.6% (0)
(171)	74		-	-	-	-	-	-	-	-	-	0
(169)	212	56	0	0	0	0	4.7%	0.9%	1.9%	2.8% (1.8%)	10.3% (1.8%)	7%
(172)	34	10	0	0	0	0	2.9%	0	0	2.9%	4.8%	2.9% (10%)
(166)	63	27	0	0	3.2%		0	3.2%	1.6%	3.2%	11.2%	4.8%
(165)	138	21	0	0	0	0	0	0	0	0.7%	0.7%	1.4%

^*^Total acute complications rate; ^†^hypopituitarism was diagnosed at follow-up.

Postoperative anterior pituitary failure is only occasionally reported in the majority of series, and menstrual cycles and fertility are usually restored if postoperative normalization of PRL is obtained in female patients. Reported complications of surgery are permanent diabetes insipidus in 2% (0–5%), meningitis in 1% (0–3%), and CSF leakage in 3% (2–5%). Other endocrine complications are usually transient, such as diabetes insipidus in 16.5% (7–28%), inappropriate antidiuretic hormone (ADH) secretion in 9% (5–14%), hypopituitarism in 2% (1–4%), namely central hypoadrenalism in 1–2%, central hypogonadism in 3–6%, and central hypothyroidism in 1–6%. The overall complication rate was higher in the first cases pointing to a learning curve. New postoperative hormonal loss has been detected in 0% of patients with adenoma diameter <20 mm and in 13.6% with adenoma diameter ≥30 mm (170). Improvement of pituitary function following surgery is relatively frequent, up to 35% in Kreutzer *et al*.’s series (169).

If less than 30–40% of patients can stop medication without recurrence of symptoms, a recurrence rate after surgery of up to 18% at 5 years can be expected (172).

Considering these results, a new role for surgery should be considered (162). Upfront TSS should be discussed at initial presentation in an individualized manner. The option of transsphenoidal resection if technically feasible should be offered to the patient with non-invasive adenoma with or without campimetric impairment. On the other hand, the invasive adenoma should be treated with DA, even in presence of severe visual impairment. In these cases, surgery should be a second-line treatment if DA are ineffective.

Another concerning point is the case of cystic prolactinomas: medical therapy is usually attempted, but poor shrinkage of cystic prolactinomas on DA therapy is not unusual, even though there are occasional reports of good results of medical treatment even in this setting (89, 174); such tumors might be treated surgically, particularly when a visual defect persists (175, 176).

It is well-known that prolactinomas in males are usually larger, more invasive, frequently with cystic components, and less sensitive to DA (8). This combination of factors suggests considering surgical treatment in this setting.

DA-induced tumor fibrosis is still a matter of discussion with conflicting reports about its real occurrence and the involved molecule (Br in most cases) (177, 178, 179).

The topic of aggressive tumors is addressed at 5.e.viii and of debulking of MP before pregnancy at 5.e.iii.

An issue to be considered is cost-effectiveness of surgery vs DA therapy: surgery is obviously associated with high upfront surgical costs due to hospitalization, but follow-up is very easy and cheap, based only on PRL sampling without routine MRI. DA treatment is conversely associated with accumulating, ongoing costs. At 10-year follow-up, surgery seems to be less costly and more effective than DA therapy (180), particularly in young patients, but there are no Italian data on this topic.

A recent meta-analysis of the available literature indicates that surgery is indeed a viable alternative first-line treatment for prolactinoma patients, especially for young microP female patients (164).

After surgery, MRI should be performed approximately after 3–4 months and further radiological follow-up strictly depends on PRL levels.

### 5.d. Radiation treatments (R 35–38)

Patients who have intolerance or resistance to DA often require surgery as a second-line approach. In such a clinical scenario, radiotherapy is usually reserved for patients after an unsuccessful surgical procedure who still have a tumor remnant resistant to DA or an uncontrolled tumor growth. Therefore, the clinical indications for radiation in patients with a PRL-secreting adenoma are, nowadays, rather limited.

The goal of radiotherapy in patients with prolactinoma is control of tumor growth. Normalization of PRL hypersecretion is an ancillary objective.

Old series on the long-term results of external beam fractionated radiotherapy typically found tumor control in over 80% of cases and normalization of PRL levels in 20–30% of the patients (reviewed in (113, 181)).

At present, the most widespread technology to irradiate pituitary tumors is stereotactic radiosurgery using gamma knife or cyberknife or proton beam, which takes advantage of using a very focused and high-energy beam of radiation to the biological target in a single fraction. Following the widespread availability of gamma knife radiosurgery (GKRS) units from the ’90s, more experience on the efficacy of GKRS in prolactinoma is slowly accruing.

A recent multicenter study collected the long-term results in 289 patients and showed tumor growth control in 95% of treated adenomas (5% had tumor progression) at last follow-up (mean 60 months up to 267 months) and normalization of PRL levels in 43% of the patients at 5 years and 54% at 8 years (182). From a clinical standpoint, another relevant endpoint of GKRS in patients with partial resistance to DA is the normalization of PRL levels while continuing medical treatment. The endocrine remission rate of combined treatment approaches 50–70% at 5 years (182, 183, 184).

The investigation of prognostic characteristics for endocrine remission after radiotherapy has been particularly difficult, probably because of the small sample size of most series. This problem has been circumvented in the multicenter study by Hung *et al*. (182). They found that higher PRL levels (namely >270 ng/mL in their series) before GKRS were the only factor independently associated with an unsuccessful outcome.

A still unresolved issue is whether DA taken concomitantly may diminish the efficacy of radiation. Conflicting results have appeared (182, 183). Besides the problem of the retrospective nature of all studies and small sample size, the degree of the resistance to DA – partial or complete – probably contributes to the different results. Given the perduring uncertainty, some authors advocate for temporary 4-week discontinuation of DA to avoid a potential factor counteracting the effects of GKRS (181), but caution should be exercised and the dangers of a possible tumor expansion after drug withdrawal should be fully balanced, checking regularly PRL levels off DA treatment.

There is a need for serial MRI (and PRL) monitoring after radiation in most patients, every 3–6 months in the first year and thereafter according to initial response for at least a few years. When normalization of PRL levels is reached, the ongoing medical treatment with DA can be slowly tapered with the aim of a definitive withdrawal in those patients with persistent normal PRL levels.

As it is already standard of care after radiotherapy to the sellar region, the pituitary function should also be monitored as appropriate to document the development of hypopituitarism and start replacement treatment promptly (71). New hypopituitarism occurred in 25% of irradiated patients in the series by Hung *et al*. (182), namely 15, 11, 15, and 6% for hypogonadism, hypoadrenalism, hypothyroidism, and GH deficiency, respectively. The onset of new hypopituitarism is mainly related to the dose at the stalk that should be maintained lower than 8–10 Gy.

The risk of rare but severe side effects after GKRS treatment seems very low, unless the patient had already received radiation therapy in the past. New visual damage was observed in 3% of patients in the series by Hung *et al*. (182). Very recent studies show reassuring data on the risk of developing neurocognitive deficits (185) or brain neoplasms (186), even though follow-up was limited to 5 and 8 years for psychometric and oncologic evaluations, respectively.

### 5.e. Special cases

#### 5.e.i. Children (R 39)

Due to rarity of prolactinomas in children, only small retrospective series are available. DA remain the first-line treatment in both microP and MP, the goals of this treatment being the restoration of normal gonadal function and tumor shrinkage (1). Br has been found to be effective in controlling PRL secretion in less than 70% of cases (187). Conversely, Cab (median dose of 2 mg/week) has been found to be effective in normalizing PRL in most treated patients (74–87%), tumor shrinkage being observed in up to 80% of cases (16, 188, 189, 190). A recent publication reported a worse response to Cab that was able to normalize PRL levels in 54% of patients only (191).

Though the incidence of adverse events of DA has not been systemically investigated in the pediatric population, these are similar to those observed in the adults. While the most common gastrointestinal effects are nausea and vomiting that may lead to DA discontinuation in 3–5% of pediatric patients, orthostatic hypotension is reported in up to 5% of them (16, 187, 188, 189, 190, 191).

DA resistance is associated with higher PRL levels and larger tumors (16). Interestingly, MEN-1 mutations seem to be an independent predictor of DA resistance in this group of patients (16), AIP mutations being less frequently found than in pediatric and adolescent patients with acromegaly/gigantism (16).

The transsphenoidal surgical approach is mainly indicated in those patients not responding or intolerant to DA treatment (1). It is worth noting that surgical approach may be more difficult in little children due to anatomical reasons (188). The recurrence rate of prolactinomas after neurosurgery seems to be higher than that described in the adult population (30% vs 20%), this observation being in line with what observed in pediatric patients with other secreting pituitary adenomas (191, 192, 193).

Few data are so far available on the effects of both radiotherapy and radiosurgery in pediatric population with prolactinomas. Salenave *et al*. observed PRL normalization in three out of four patients who received radiotherapy due to DA resistance and after surgical failure (16). Yang *et al*. described three patients who underwent GKRS, all showing >90% reduction of tumor size and symptoms remission and no new onset of hypopituitarism being diagnosed during the follow-up (191).

GH treatment is rarely necessary because DA therapy is generally sufficient to normalize GH deficiency and to restore growth (16). In children with tumor remnant needing GH replacement therapy, a tighter follow-up is needed.

#### 5.e.ii. Women requiring contraception or sex hormone replacement therapy (R 41–43)

Attempts to link the progression of PRL-secreting adenomas to oral contraceptive use have recently proved negative and many studies have excluded this correlation (150, 194, 195, 196).

Women who do not wish to conceive should practice a reliable method of contraception. The safety of estroprogestinic pill was demonstrated in women with microP whose cycles were restored by DA treatment (151). In these cases, estroprogestinic pill may be considered as the only treatment of the disease. At variance, in DA nonresponder MP patients, the persistence of amenorrhea, symptoms of hypogonadism, and the control of tumor growth are an issue. Since ERT may cause a decrease in the efficacy of DA, the evaluation of PRL secretion before and after the start of replacement therapy and the careful measurement of adenoma size by MRI over the first year are warranted, while continuing DA therapy (151). In these cases, the lack of tumor shrinkage may be acceptable as long as no tumor growth is observed. However, further prospective studies including large numbers of patients are needed to validate this practice.

#### 5.e.iii. All about pregnancy from ovulation to breastfeeding (R 44–57)

##### Before conception

Approximately 90% of women with hyperprolactinemia will start ovulating within a few weeks after the start of DA treatment (113).

Neurosurgical option for microP and enclosed MP to avoid DA and for MP in DA-resistant patients (mostly if close to optic pathways) to avoid symptomatic enlargement during pregnancy should be discussed with the patient (162, 197).

It is recommended to plan pregnancy. The treatment, either DA or surgery, should lower PRL levels to allow conception and in case of MP should shrink tumor within sellar limits in order to minimize the risk of enlargement during pregnancy. It is recommended to perform MRI prior to conception in MP to confirm the shrinking efficacy of DA treatment.

In microP, any attempt at conception should be postponed at least until the first DA-induced menstrual cycle. Successful pregnancy is common after DA treatment (197, 198, 199), but gonadotropins can be used to revert hypogonadotropic hypogonadism if DA are unsuccessful.

##### During pregnancy

In normal pregnancy, pituitary gland volume is increased by about 70% and PRL levels increase. PRL does not reliably reflect an increase in tumor size and thus is not useful for clinical assessment (197). An increase in tumor size correlates better with clinical symptoms, such as headaches or visual disturbances.

Br and Cab are classified as pregnancy risk factor B (animal reproduction studies failed to demonstrate a risk to the fetus and there are no adequate and well-controlled studies in pregnant women) and have been shown to cross the placenta (200). Accordingly, DA should be stopped at confirmation of pregnancy (if not already discontinued before). In this way, there will be exposure to DA for only about 3–4 weeks of gestation.

**Microprolactinoma**. The risk of significant tumor growth during pregnancy is less than 3% (197, 198). The patient should be followed clinically during pregnancy (197). Visual field testing should be carried out in patients who develop symptoms and MRI when intervention may be indicated.

**Macroprolactinoma**. The management and prognosis of DA-treated MP during pregnancy remains unclear. Few case series have been reported but the risk of symptomatic tumor enlargement (with headache and visual loss due to pressure effect on the optic chiasm) is high (197, 198, 201): up to 20–30% of women with no prior surgery or irradiation and in the range of 5% of those previously operated on or irradiated (197). In a recent two-center, retrospective, observational study, a total of 85 viable pregnancies were observed in 46 patients with MP (201). Tumor growth-related symptoms were identified 12 times in 9 patients, including 3 cases of apoplexy. Restarting, changing, and/or increasing DA treatment was effective in 10 cases. Emergency surgery had to be performed twice (due to pituitary apoplexy). Patients with tumor progression tended to present with larger tumors after initial treatment and before pregnancy, whereas adenoma size at diagnosis did not seem to be a significant predictive factor (201). The obstetrical outcomes were comparable to the general population.

The patient with a small intrasellar or inferiorly extending macroadenoma can probably be managed as those with microadenomas (197). Close clinical monitoring should be undertaken with formal visual field testing during each trimester (197). The continuation or reinstitution of therapy should be considered in the case of large pituitary tumors particularly if the adenoma is in close vicinity to the optic chiasm or signs of tumor expansion develop during pregnancy (197, 198, 202). Due to its higher efficacy and better tolerability, Cab is preferable in these cases (202).

In a small proportion of those cases, tumor enlargement reflected apoplexy, which may require an entirely different management course according to specific guidelines, including surgery and hormone replacement for hypopituitarism. MRI may be very helpful in distinguishing between hemorrhage from a tumor vs simple tumor enlargement.

Since a slight increase in risks of stillbirths and neonatal deaths was shown with first trimester Gd exposure (203), it is recommended not to use Gd throughout pregnancy, whereas MRI can be performed safely after the fourth month only for particular circumstances, such as uncontrolled worsening disease with severe headache, visual impairment, or cranial nerve palsies.

Any surgical procedure during pregnancy results in a 1.5-fold increase in fetal loss in the first trimester and a 5-fold increase in the second trimester (197). Emergency surgery for sellar/parasellar lesion caused abortion in 1 out of 6 cases reported by Zoli *et al*. and in 2 out of 25 in their systematic literature review (204).

The risks of congenital malformations during the first trimester and those of premature birth during the third trimester led to a preference for the second trimester to consider surgery. Although limited, surgical risks remain difficult to assess (205). In the third trimester, preterm delivery should be considered (202).

In conclusion, the management should be individually tailored, taking into account the severity of symptoms at presentation, disease progression, and surgical expertise available, with a fine balance between the risks for the mother and the risk of surgery for the baby (206).

##### Delivery

If there are not obstetric contraindications, vaginal delivery can be safely performed in women with microadenoma (201).

The risk of apoplexy should be carefully considered together with the obstetrician when evaluating the modality of delivery, vaginal or cesarean, in the patient with symptomatic macroadenoma (201).

##### Obstetrical outcome

Data regarding less than 6-week exposure to DA have been reported in more than 6000 and 1000 pregnancies for Br and Cab, respectively, with no increase in spontaneous abortions, preterm deliveries, multiple births, or congenital malformations compared to general population (152). Namely, malformations were reported in 3.4% of Cab exposed pregnancies vs 6.4% in the general population (207).

Maternal and fetal outcomes in Cab-induced pregnancies are reassuring also in recent series (199, 208, 209). On the contrary, higher miscarriage rate was reported in those patients treated with Cab throughout pregnancy for tumor volume increase (209).

There are just over 100 women reported to have used Br throughout gestation (210) and no abnormalities in their infants were found, except for two cases with minor malformations. Use of Cab throughout gestation is described in 15 women (211), with 1 preterm delivery and 1 intrauterine death.

##### Breastfeeding

Although suckling stimulates PRL secretion in normal women, there are no data to suggest that breastfeeding can cause tumor growth (208). On the contrary, nursing caused neither an increase in PRL levels nor headaches or visual disturbances, which would suggest tumor enlargement (197). Accordingly, breastfeeding should be allowed in patients with uneventful pregnancies, postponing the possible restart of DA treatment (152, 211).

##### After pregnancy

A retrospective study conducted in two Belgian academic centers including 73 patients (54 microPs and 19 MPs) reported normal PRL levels without medication in more than 40% (both microP and MP) of women previously diagnosed with prolactinoma and followed-up until a median of 22 months after pregnancy and lactation (212).

A recent series confirmed that approximately a quarter of women with prolactinoma will have normal PRL levels without treatment after pregnancy (199). The likelihood of remission was associated with a smaller initial adenoma size, lower PRL levels at diagnosis and postpartum, and older maternal age (199, 212). The number of pregnancies per woman as well as breastfeeding and its duration did not influence remission rate (198, 208, 212). In case of remission, clinical and hormonal follow-up at 6 months after delivery and yearly thereafter has been recommended (202).

#### 5.e.iv. Postmenopausal women (R 58–59)

Most MP patients in postmenopausal age show response to DA therapy with PRL level normalization and adenoma shrinkage (115, 152).

Menopause may have a beneficial effect on the natural history of prolactinoma patients (152). In some studies, untreated patients with microP during menopause had spontaneous normalization of PRL levels and disappearance of the tumor (213). As a further confirmation of the favorable impact of menopause, the relapse of hyperprolactinemia after DA withdrawal in microP is nearly 30% in postmenopausal patients (far lower than the 70% reported in premenopausal women) (152).

Serum PRL levels detected 6–12 months after DA withdrawal are a useful predictor of subsequent trend that can guide clinical practice both in microP and MP. Tumor progression may occur in nearly 7% of patients with microP and PRL levels may reincrease in MP. Therefore, a regular follow-up is required in patients showing increasing hyperprolactinemia that should be treated according to standard clinical practice (213).

#### 5.e.v. Women with personal or familial history of breast cancer (R 60)

Experimental data showed that PRL receptors are expressed on breast cancer cells (214) and that PRL does not induce breast carcinogenesis but has a positive effect on breast cancer progression in animals (215).

Pathophysiologic data showed that PRL levels in the higher quartile of the normal range are associated to increased risk of breast cancer and invasiveness, particularly in postmenopausal women (216), and that the addition of PRL levels (even within the normal range) improves the predictive value of traditional risk factors for breast cancer (217).

Notwithstanding these experimental data and pathophysiologic premises, epidemiologic studies did not show any increase of breast cancer, either in patients with prolactinomas or idiopathic hyperprolactinemia from Netherlands, Sweden, and Denmark (218) or in patients treated with antipsychotic drugs (219).

We can speculate that hyperprolactinemia-induced hypogonadism might have a protective effect against breast cancer.

#### 5.e.vi. Patients with a psychiatric disease (R 61–63)

It is well-known that treatment of several psychotic disorders is based on drugs that act as dopamine antagonists. As a consequence, the treatment with antipsychotic drugs might cause hyperprolactinemia. The workup of a suspected drug-induced hyperprolactinemia has been already discussed (*at 4.b*.).

Treatment of symptomatic neuroleptic-induced hyperprolactinemia aims to restore menses and sexual function without causing a flare-up of psychosis. Accordingly, the management of hyperprolactinemia in psychiatric patients needs a tight collaboration between endocrinologists and psychiatrists, with the joint evaluation of individual drug need and selection. Different approaches should be considered in symptomatic patients (79). Whereas the use of DA to treat neuroleptic-induced hyperprolactinemia might cause worsening of psychiatric symptoms, as reported in a few cases (220), on the other hand, small series did not report exacerbation of psychosis when small doses of DA were used (221). In addition, estroprogestinic treatment may be used to treat hypogonadism in women, whereas the use of testosterone in men should be approached with extreme caution.

The association between psychotic disorders and prolactinoma is rare. A multicenter retrospective study collected data from 1987 to 2017 of 18 patients affected with both severe psychotic disorder and MP (222). The question raised by this retrospective study is whether DA may reduce the efficacy of antipsychotic drugs in psychiatric patients affected with MP. Each patient required both antipsychotic drugs for her/his psychiatric disease and DA for treating the MP. Among the psychiatric relapses requiring admission in psychiatric units that were observed in nine patients during the follow-up period, none could be certainly imputed to the use of DA. Combined treatment with antipsychotic drugs and DA shrank MP in a high percentage (87.5%) of cases, even though the magnitude of tumor size reduction was smaller than that observed in patients without a psychiatric disease. Only 25% of patients reached normal PRL levels. Although a strong relationship was not demonstrated, the efficacy and safety of DA in a psychiatric patient affected with a prolactinoma should be individually evaluated.

#### 5.e.vii. Treatment of osteoporosis (R 64)

The treatment of prolactinoma with DA normalizes serum OC levels, a parameter of bone formation, decreases NTX levels, a bone resorption marker (39), improves BMD, and prevents further bone loss by reducing PRL levels and by restoring the functionality of the gonadal axis (39, 43, 46, 223, 224).

There are no prospective controlled studies regarding the effect of the treatment of hyperprolactinemia on the outcome of fracture risk. In a meta-analysis, vertebral fractures were reported in 46% of untreated female patients vs 20% of patients on Cab (OR: 0.29; 95% CI: 0.10–0.78) and in 67% of untreated male patients vs 26% in Cab-treated patients (OR: 0.18; 95% CI: 0.03–0.94), with no difference between eugonadal and hypogonadal men (*P*  = 0.8) (225). This suggests a beneficial effect of DA treatment on fracture risk. Patients with prolactinoma had a signiﬁcantly higher relative risk of fractures compared to normal controls before the diagnosis (RR = 1.6) but not after diagnosis (RR = 1.2) (50). However, the prevalence of vertebral fractures remains higher in men treated for prolactinomas than in the age- and gender-matched control population (48).

Even though normalization of PRL concentrations by DA therapy can improve BMD and can reduce the risk of fracture, it may be necessary to treat specifically osteoporosis with the same therapeutic options as in the general population in addition to the treatment for hyperprolactinemia. Furthermore, in light of the recent evidence of a detrimental direct effect of hyperprolactinemia on bone, osteoporosis and the risk of fracture might change the indications for treatment with DA, especially in postmenopausal women (226) and in patients with iatrogenic hyperprolactinemia, but more data are needed.

#### 5.e.viii. Resistance to treatment and aggressive disease from definition to multimodal treatment (R 65–69)

A universal consensus on the definition of DA resistance is still lacking, even though the widely accepted definition is a failure to normalize PRL on maximally tolerated doses of DA and a failure to achieve at least 50% tumor size reduction (227). The maximally tolerated doses vary among patients: doses up to 12 mg weekly and 30 mg daily were anecdotally reported for Cab and Br, respectively (113, 120, 228). In common clinical practice, the mean maximum dose of Cab is around 4 mg per week (109). Even though no agreement exists about the minimum duration of treatment to define resistance, it was suggested at least 6 months on the highest tolerated DA dose (229).

A subset of individuals with prolactinomas will exhibit a varying degree of resistance to DA (109, 228). Some patients may respond poorly to one DA but well to another or individuals may respond well initially and later become drug resistant and show a recurrence of the disease. Cases of ‘selective’ resistance have been reported in a few patients in whom the drug induces discordant PRL-lowering and tumor size-reducing effects.

Resistance is more frequent in cases of MP, invasive tumors, and male patients. Other factors are very young age, cystic, hemorrhagic and/or necrotic components (before the start of pharmacological treatment) inside the tumor, and genetic predisposition to develop pituitary tumors such MEN-1 or AIP mutations (228, 230, 231).

The estimated prevalence of resistant prolactinomas is approximately 20–30% for Br and 10% for Cab (228). In poor responders, Cab doses higher than 3.5 mg weekly could achieve a controlled PRL within 1 year (120). In resistant prolactinomas, PRL normalization could be achieved in 26% of patients with some degree of tumor shrinkage in 52% of them by receiving at least daily Cab therapy (>3.5 mg/week) (109).

In patients with Cab-resistant prolactinomas, a Br trial was anecdotally reported as a safe and well-tolerated alternative (110).

Patients partially resistant to medical treatment may benefit from neurosurgery, even though tumor resection is likely incomplete. Surgical debulking may improve hormonal control with lower postoperative doses of Cab (109, 166). Radiotherapy should be offered to patients who still do not respond to DA treatment after unsuccessful surgery (228). Surgery can be repeated in resistant/aggressive cases.

PRL-secreting tumors frequently express somatostatin receptors (SSTR), particularly SSTR5 and SSTR1 and to a lesser extent SSTR2 (232). Very preliminary data support that in some patients with DA-resistant MP, the addition of octreotide LAR or pasireotide LAR to ongoing Cab therapy may result in significant reductions in tumor volume and PRL levels (233, 234).

Neuroradiological monitoring of resistant prolactinomas is performed on a case-by-case basis. Up to 30% of aggressive MPs do not respond with significant shrinkage to Cab (228).

In a minority of patients with complete resistance to DA treatment and unsuccessful surgery and radiation therapy, the tumor may show unrelenting and rapid growth (1, 235). In rare cases, patients may develop the presence of distant metastases, either through dissemination into the CSF or to other organs (pituitary carcinoma). The therapeutic options in patients with an aggressive prolactinoma or a pituitary carcinoma are rather limited. The first option is chemotherapy with the alkylating agent temozolomide. In a recent survey of the European Society of Endocrinology that collected the data on 165 patients who had been treated with temozolomide (236), prolactinomas accounted for 20% of all aggressive pituitary tumors and for 37.5% of all pituitary carcinoma. A positive response to temozolomide, defined as complete, partial, or stable disease, was reported in 79% of all patients and was independent of tumor type (hormone active or inactive).

Patients who had progression of disease during temozolomide treatment or who had recurrence of disease after an initial response had a grim prognosis. Therapeutic options in this setting are very limited. Other chemotherapeutic agents or immunotherapy have been tried but the results have been poor or short-lived. Peptide receptor radionuclide therapy is another last resort treatment for such patients. However, few cases of prolactinomas are reported with mixed results (237).

### 5. f. Therapeutic strategy possible shift to surgery as first-line option (R 16–20, 25–27, 33)

Medical therapy with DA is still considered the first therapeutic option of hyperprolactinemic states (1) because it can obtain restoration of eugonadism and tumor control with disappearance of neurological and ophthalmologic symptoms in most patients. It is however unable to attain the remission of the disease in most patients (143). On the contrary, neurosurgery, still considered as ancillary to medical treatment to be employed only in resistant or aggressive cases, thanks to its technological improvement could be a viable alternative, because it is able to obtain disease remission in the majority of patients with enclosed adenomas, with a very low risk of long-term complications (2, 165, 166). Neurosurgery can thus be regarded as the first option in selected clinical settings.

In centers with experienced neurosurgeons, the possibility of cure by upfront surgery vs life-long DA therapy should be discussed by the endocrinologist and the neurosurgeon with all patients in an individualized manner. The patient should be informed that drug therapy is safe and effective, can obtain restoration of eugonadism, can cause disappearance of neuro-ophthalmologic symptoms, and lead to tumor shrinkage in most, without causing any loss of pituitary function, but it is often lifelong. On the other hand, surgery in selected cases offers the real possibility to cure the disease.

Surgery should be particularly considered for non-invasive microP in which a very high cure rate (even over 80% in the best hands) can be anticipated (2) and in circumscribed MP given the high cure rates, comparable to microP. The will of an informed patient is an important indication for first-line TSS (1, 172, 238).

In large prolactinomas with invasive character, DA therapy should be initially preferred due to the low cure rate and the increased risk of surgery in this setting (165, 169, 239). Whenever DA treatment does not revert neuro-ophthalmologic symptoms quickly, the patient should be rapidly referred to neurosurgery.

Prolactinomas in male patients may behave more aggressively and show an increased likelihood of resistance to DA treatment. Hence, a giant/macroadenoma, mostly in a male patient, is a strong indication to a tight follow-up and may swing the pendulum in favor of surgery, provided that a trial of DA treatment had no effect on tumor-related symptoms (240).

Young age is another important aspect to be considered in proposing surgical treatment to minimize the cumulative dose of DA (136, 180).

The patients’ wish to become pregnant is a re-enforcing argument if surgery is considered. In microP, surgery offers a high likelihood of cure and the opportunity to become pregnant without DA medication. Surgery should be of course performed by a skilled operator, to minimize the risk of pituitary damage and fertility impairment. In MP, if the patient is not willing to wait for DA results, pre-pregnancy debulking significantly reduces the risk of symptomatic enlargement during pregnancy (165, 241).

**Box 1. Tips and tricks**

1. Serum prolactin (PRL) should be measured only in specific clinical settings, such as menstrual disturbances, erectile dysfunction, loss of libido, infertility, or imaging showing a lesion in the hypothalamic-pituitary region.
2. The insertion of an i.v. catheter 15–20 min before sampling for PRL assay in the diagnostic phase is a simple and practical tool for the confirmation of diagnosis in cases of mild hyperprolactinemia (<80–100 ng/mL).
3. Try to identify the cause of hyperprolactinemia, including iatrogenic and other causes different from PRL-secreting tumors (see text for details).
4. Do not start treatment with dopamine agonist (DA) drugs without a diagnosis and a pituitary MRI if appropriate.
5. The magnitude of PRL levels in macroadenomas often enables to differentiate between ‘true’ prolactinomas and ‘pseudoprolactinomas’ (see text for details).
6. The management should be guided by clinics and not only by ‘numbers’.
7. Patient’s wishes should be taken into account in the management.
8. Do not forget hypopituitarism.
9. Single PRL measurement is adequate during DA treatment.
10. In patients on chronic DA treatment, no particular timing is required for PRL sampling.
11. During intercurrent illness or scheduled hospitalization, chronic DA treatment should be continued.
12. Alert the patient and caregivers about the possible DA-induced impulse control disorder.
13. Advice female patients to plan pregnancy and provide easily accessible medical support to pregnant women.
14. The choice about delivery and breastfeeding should be shared with the endocrinologist.
15. In complex cases, do not hesitate to refer patient to experts: aggressive tumors should be treated by a pituitary multidisciplinary team.

Concerning aggressive prolactinomas, it is conceivable that a combination of the different treatment modalities (DA, surgery, radiotherapy, and temozolomide) will afford the best results, but firm data are lacking on this topic.

Finally, it is worthwhile considering that prolactinomas are the most frequent pituitary tumors and microPs account for the majority of them. The indication for surgery should thus take into account the large prevalence of the disease and the need to refer patients to high-volume neurosurgical centers with expertise in pituitary surgery.

## 6. Conclusions and perspectives

We are facing a paradigm change in the management of prolactinomas. Both DA and neurosurgery might now be regarded as a first-line therapeutic option in certain clinical and neuroradiological scenarios, such as a non-invasive adenoma, regardless of size. In this clinical situation and when feasible, a shared decision should be implemented with full provision of information about benefits and risks of both treatment options, taking into account patient’s preferences and values. Whereas most endocrinologists are potentially able to manage DA treatment, reported data clearly show that the best surgical results are obtained by neurosurgeons with a high caseload of pituitary operations (at least 50 pituitary operations per year) (242, 243, 244).

An integrated network among hub and spoke centers able to manage even complex cases in multidisciplinary teams should be implemented.

## Declaration of interest

The following authors disclose competing interests in the past 2 years: Alessandro Bozzao, Marco Caputo, Laurence Kaznelson, Giovanni Lasio, Marco Losa, Davide Milani, Catalina Poiana, and Michele Zini report that they do not have any relevant financial relationships with any commercial interests. Maria Rosaria Ambrosio reports registration fees for scientific meetings from Ipsen, Novartis, Pfizer, and Savio Pharma. Roberto Attanasio reports registration fees for scientific meetings from IBSA, Pfizer, and Novartis. Claudia Battista reports registration fees for scientific meetings from Ipsen and Eli Lilly. Enrica Ciccarelli reports registration fees for scientific meetings from Novartis. Renato Cozzi reports that he has been a member of the Advisory Board of Novartis, received research fees for scientific meetings or oral presentations from Ipsen, Italfarmaco, and Novartis. Laura De Marinis reports that she has been Principal Investigator for clinical trials for Novartis, Ipsen, Pfizer, and Chiasma. Ernesto De Menis reports registration fees for scientific meetings from Novartis, Ipsen, and Pfizer. Marco Faustini Fustini is a member of the Advisory Board of Pfizer. Franco Grimaldi received honoraria and had roles as consultant or advisor from Ipsen, Pfizer, Novartis, Advanced Accelerator Applications – AAA. Andrea Lania reports that he is a member of the Advisory Board of Novartis, received research grant support from Novartis, and registration fees for scientific meetings from IBSA, Pfizer, Novartis, and Shire. Francesco Logoluso has been a member of the Advisory Board of Novartis and received registration fees for scientific meetings from Ipsen, Pfizer, Novartis, IBSA, and Shire. Anton Luger: receipt of honoraria and/or consultation fees from Ipsen, Merck Serono, Novartis, Pfizer, Sandoz and grants or research support to the Clinical Division of Endocrinology and Metabolism, Department of Medicine III, University of Vienna from Ipsen, Novartis, Pfizer. Pietro Maffei reports that he has been a member of the Advisory Board of Novartis and Pfizer, received research fees for scientific meetings or oral presentations from Pfizer, Novartis, and Ipsen. Maurizio Poggi reports registration fees for scientific meetings from Novartis, Eli Lilly, and Ipsen.

## Funding

This work did not receive any specific grant from any funding agency in the public, commercial, or not-for-profit sector.
